# Incidence of Gastric Cancer in Marrakech and Casablanca, Morocco

**DOI:** 10.1155/2015/704569

**Published:** 2015-10-21

**Authors:** Brittney L. Smith, Mouna Khouchani, Mehdi Karkouri, Audrey J. Lazenby, Katherine Watkins, Ali Tahri, Abdel-Latif Benider, Shireen Rajaram, Amr S. Soliman

**Affiliations:** ^1^Department of Epidemiology, College of Public Health, University of Nebraska Medical Center, Omaha, NE 68198, USA; ^2^Department of Oncology, Center Hospital University Mohammed VI, 40080 Marrakech, Morocco; ^3^Department of Medicine and Pharmacy, Cadi Ayyad University, 40000 Marrakech, Morocco; ^4^Department of Pathology, Center Hospital University Ibn Rochd, 20100 Casablanca, Morocco; ^5^Department of Pathology and Microbiology, College of Medicine, University of Nebraska Medical Center, Omaha, NE 68198, USA; ^6^Department of Health Promotion and Disease Prevention, College of Public Health, University of Nebraska Medical Center, Omaha, NE 68198, USA

## Abstract

Gastric cancer is the fifth most common cancer globally with over 70% of new cases occurring in developing countries. In Morocco, oncologists in Marrakech suspected higher frequency of gastric cancer compared to Casablanca, a city 150 kilometers away. This study calculated age-specific, sex-specific, and total incidence rates of gastric cancer in Marrakech and was compared to the Casablanca population-based cancer registry. Using medical records from Center Hospital University Mohammad VI and reports from 4 main private pathology laboratories in Marrakech, we identified 774 patients for the period 2008–2012. Comparison of rates showed higher age-specific incidence in Marrakech in nearly all age groups for both genders. A higher total incidence in Marrakech than in Casablanca was found with rates of 5.50 and 3.23 per 100,000, respectively. Incidence was significantly higher among males in Marrakech than males in Casablanca (7.19 and 3.91 per 100,000, resp.) and females in Marrakech compared to females in Casablanca (3.87 and 2.58 per 100,000, resp.). Future studies should address possible underestimation of gastric cancer in Marrakech, estimate incidence in other regions of Morocco, and investigate possible risk factors to explain the difference in rates.

## 1. Introduction

Cancer is an emerging global health problem in developing countries. While developed countries have seen increasing incidence rates over the past several decades, developing countries have begun to experience the rising trend of cancer incidence and mortality [1]. Breast and cervical cancers are the most common cancers in women and lung cancer is the most common cancer in men in developing countries [2].

Developing countries with the highest rates of cancer are mostly in Asia and South America, with incidence rates greater than 260.4 per 100,000. Africa, a continent that is much slower in its development, only sees about 94.8–127.5 cases per 100,000, with relatively higher rates in Northern Africa. Morocco, a country with development and epidemiological transitions growing faster than most other countries in Africa, falls into the relatively higher incidence rates within Africa [2].

Gastric cancer is the fifth most common cancer globally. Over 70% of new cases are from developing countries and half of all cases of gastric cancer are in Eastern Asia [3]. Globocan statistics of 2012 [3] show gastric cancer rates as highest in Asia and lowest in Africa. Globocan [3] estimated an age standardized rate of gastric cancer in Morocco as 5.1 per 100,000 for males and 3.0 per 100,000 for females. As a reference, the world age standardized rate of gastric cancer is 17.4 per 100,000 in men and 7.5 per 100,000 in women for 2012 [3]. In recent years, rates of gastric cancer have been declining in most countries in Europe, Asia, and North America; however, with limited data from Africa it is difficult to make this generalization to African countries as well [4]. Impressions from local oncologists in Marrakech, Morocco, suggest that gastric cancer may be seen more frequently in Marrakech than other regions in Morocco. Thus, this study was conducted to determine the age- and sex-specific and total crude incidence rates of gastric cancer in Marrakech and compare them to the rates in Casablanca, the largest city with a population-based cancer registry in Morocco.

## 2. Methods

### 2.1. Study Setting and Population

This study was conducted at the Center Hospital University Mohammad VI (CHU) in Marrakech, Morocco. This hospital is the largest of the 4 hospitals in Marrakech and the only public hospital for diagnosis and treatment of cancer in the city. The hospital serves patients from the city of Marrakech (population of 1.063 million in 2012) and its surrounding region, Marrakech-Tensift-Al Haouz [5]. A limited number of patients also come from the neighboring regions of Souss-Massa-Drâa and Tadla-Azilal. In 2012, the hospital diagnosed and/or treated 1,573 cancer patients who were covered by government health insurance. A majority of the patients seen at the hospital were from low- and middle-income socioeconomic levels of this community. The smaller portion of the patients who are from upper middle-income and upper socioeconomic levels seek care at the few private clinics and hospitals in Marrakech. Due to possible long waiting times for histopathologic examination of biopsy and resection specimens in government hospitals in Morocco, government health insurance allows patients to have the histopathologic examination done at private pathology laboratories.

Administratively, Morocco is divided into 15 regions. Cities of residence of patients found in the hospital medical records in this study included 47 cities, of which 32 were within either the region surrounding Marrakech, the Marrakech-Tensift-Al Haouz region, or the nearby regions of Souss-Massa-Drâa or Tadla-Azilal. Of these 32 cities, 22 were reported as being from the region surrounding Marrakech. From this information and the referral patterns of all cancer patients seen at the hospital, we believe that most patients who come to the hospital in Marrakech were from one of these 3 regions; thus, these regions became our study area. We were unable to get a reported residence from the patients collected from private pathology labs, because it is not part of the standard pathology form, and therefore had to assume their referral patterns matched that of the hospitals and patient's residence fell within one of the three regions.

### 2.2. Data Collection

The study identified 327 new gastric cancer patients diagnosed and/or treated in CHU in the 5-year period (2008–2012). Logbooks and medical records of gastric cancer patients were retrieved and abstracted. A manual search of the oncology records was also done to identify additional patients not recorded in the logbook.

Information for age, sex, place of residence, clinical diagnosis, and histopathologic features of the tumor was abstracted. Clinical and pathological information including symptoms and signs at presentation, endoscopic and abdominal CT scanning results, histopathologic type of tumor, tumor differentiation, presence of intestinal metaplasia, and any results on* H. pylori* infection was collected.

To supplement the pathology information of the patients identified from the study hospital and identify other patients who were treated at other healthcare facilities, we reviewed the records of the 4 main private pathology laboratories in Marrakech. The review of the records of gastric cancer patients diagnosed in these laboratories during the period of 2008–2012 revealed 470 additional gastric cancer patients. Information obtained included name, pathology reference number, age, sex, histopathologic type, differentiation, and* H. pylori* infection (if noted).

The population data used for calculating the incidence rates of Marrakech was obtained from the 2004 census data [5]. Population data for each region, province, and city in Morocco was obtained. For each city, the age-specific population was recorded. Percent growth change and natural population increase rates were used to estimate the population in Marrakech and the surrounding regions for the years in which data was abstracted from the medical records and pathology reports.

The incidence rates of Casablanca were obtained from the most recent editions of the population-based cancer registry of the Region of Grand Casablanca for years of 2004 [6], 2005, 2006, and 2007 [7]. This registry information is collected and stored within the Oncology Center at the Centre Hospital Ibn Rochd, within a specific data processing department. The Casablanca registry data covers cancer patients in all clinics, laboratories, and government hospitals in Casablanca.

### 2.3. Data Management

Since patient data was gathered from medical records as well as pathology reports from private laboratories, having data twice for one patient was possible. Thus, to eliminate duplicates, the pathology reference number used in the laboratories and recorded in the medical records was used to identify and remove duplicated data for one patient. The name of the patient was also used to verify possible duplicate records. To prepare for data analysis, medical records and pathology reports were merged into a final database and a unique study ID was given after stripping the names of patients.

**Figure 1:**
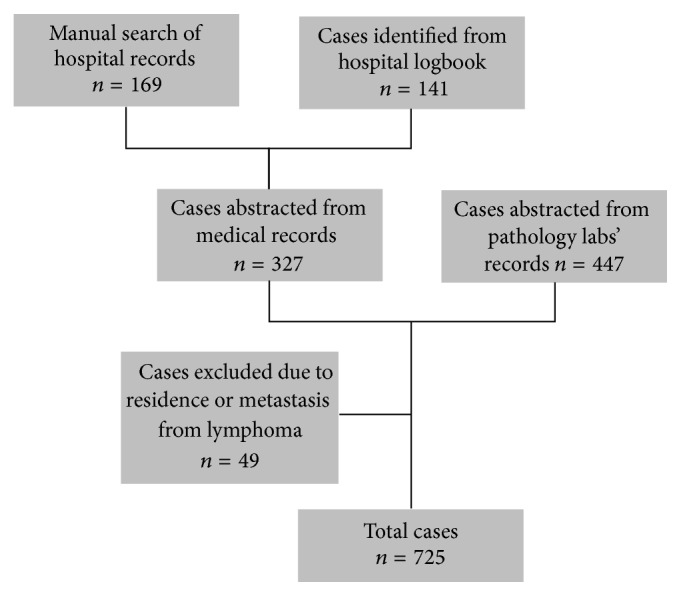
Sources of gastric cancer patients during the study period, 2008–2012, in Marrakech. Flow diagram of identification of patients from the hospital medical records and private pathology laboratory reports.

After removal of duplicates, we were able to identify 774 patients of gastric cancer in 2008–2012 of which 447 were found in the reports of 4 large pathology laboratories and 327 from medical records of the hospital. After review of records, 49 patients were excluded because of reported residence outside of the three 3 regions defined as the at-risk population surrounding Marrakech or because of histopathologic diagnosis of stomach lymphoma, which was not included in the incidence rates from the Casablanca registry (Figure 1).

To obtain a total 4-year incidence rate for Casablanca, the sum of the total number of cases reported for each year in the registry was divided by the sum of the population for each corresponding year. At the time of publication Casablanca only had registries for a 4-year period and thus the incidence reported is a 4-year incidence rate.

### 2.4. Statistical Analysis

Descriptive statistics and incidence rate analyses were completed using SAS (Version 9.3, SAS Institute, Cary, NC). Because the last available census data from Morocco was 2004, it was necessary to estimate the population of the 3 regions surrounding Marrakech for the study years (2008–2012). Natural population change since the time of the census was determined by applying the annual growth rate to the census data to estimate the population during the study years [8]. These estimates were used as the population denominator to calculate all incidence rates. Additionally, in order to compare rates from Marrakech to rates from Casablanca, age-adjusted incidence rates were calculated for 8 age categories [9]. The 8 categories, determined by those given in the census information from Morocco, were <19, 20–24, 25–34, 35–44, 45–54, 55–64, 65–74, and >75. Data from the cancer registry in Casablanca had been standardized by using the WHO world population. Therefore, we used the WHO world (2000–2025) standard population, adjusted to match the 8 age categories, to calculate age-adjusted incidence rates in Marrakech. Incidence rate ratios and their corresponding 95% confidence intervals were calculated to determine differences in rates from Marrakech to rates from Casablanca.

## 3. Results

The median age of all patients was 60 with a range of 19–94. For males, the median was 62 with a range of 19–94 and, for females, median was 58 and range was 23–88. Most (79%) patients reported being from a city that was considered an urban city center by the Moroccan census information. Patients were clinically diagnosed with gastric cancer by both endoscopy and CT scan (Table 1). The vast majority (90%) of patients had histopathologic diagnosis of gastric cancer by biopsy; few (15%) were diagnosed by resection, including those that had both biopsy and resection. Adenocarcinoma represented 83% of the histological types of gastric cancer; carcinoma and gastric intestinal stromal tumors represented 13% and 3%, respectively (Table 2).

Age-specific incidence rates for all patients, males, and females for Marrakech and Casablanca are presented in Tables 3, 4, and 5, respectively. In nearly all age ranges, rates of gastric cancer in Marrakech were higher than the rates in Casablanca. The exceptions were all patients, male and female patients under 19 years; all patients aged 20–24 years; and all patients aged 45–54 years. In males, in nearly all age ranges, the rate was significantly higher in Marrakech. However, in females, only the age ranges 45–54, 55–64, and over 75 had rates that were significantly higher in Marrakech.

The incidence rates of Marrakech for 2008–2012 and Casablanca for the years available from their registry data, 2004–2007, are presented in Table 6. The rates of gastric cancer in Marrakech for all patients, males, and females were 5.50, 7.19, and 3.87 per 100,000, respectively. All were significantly different than the rates in Casablanca.

## 4. Discussion

This study revealed the following interesting observations. First, age-specific incidence rates of gastric cancer were higher in Marrakech in nearly all age groups for both males and females compared to respective rates in Casablanca. Second, when comparing total incidence rates by sex, males in Marrakech had a significantly higher rate than males in Casablanca. Amongst females, while the rates between the two cities were statistically significant, the difference was not as prominent as that of the males. Finally, the majority of the residence of patients found in the medical records was concentrated in the 3 regions surrounding Marrakech.

With the exception of a few age groups, Marrakech had higher age-specific incidence rates of gastric cancer than Casablanca. Rates appeared higher in Casablanca in the “under 19” age group, because the Casablanca cancer registry included pediatric cancer cases, while the patient records of cancer in Marrakech did not. Gastric cancer is known to be a cancer of elderly populations, so this does significantly impact the results found [10].

The variation of rates between sexes is not altogether surprising as gastric cancer is more common in males than females [3]. However, the results may be an underrepresentation of the true rate for females. Many patients who visit the hospital in Marrakech travel many miles across rough geographic terrains and, due to travel costs or gender and cultural norms in Morocco, women may simply seek care less often than men. In many developing countries, the presence of gender inequalities combined with factors affecting health behaviors can limit a woman's ability to make the decision to seek care [11]. Compared to the nearby country of Tunisia, women in Morocco sought health care less often, due to geographic obstacles that make traveling difficult [12].

Observing that the majority of gastric cancers were from the region of Marrakech and the 2 surrounding regions is a reasonable finding because CHU in Marrakech is the only public cancer hospital serving Marrakech and the surrounding regions. While all patients in this study were from 3 regions surrounding Marrakech, there is a possibility that other patients from less populated regions might have been missed in this study because of not seeking medical care in Marrakech. Symptoms of early onset gastric cancer are similar to other nonmalignant gastrointestinal conditions; thus, patients are not likely to travel far to seek care until symptoms become severe [13].

Approximately 25% of all cancer, globally, is linked to infectious agents [14]. Previous studies assessing patterns of gastric cancer reported low rates of the disease in Africa compared to rates in other countries, despite widespread infection by* H. pylori* in Africa [4, 15, 16]. However, these same studies also noted limited availability of cancer registries in Africa, which limits the ability to generate true incidence and prevalence rates. This study is the first to assess incidence of gastric cancer in Marrakech, allowing for comparison within the country to identify rate differences and future investigation of risk factors in relation to geographic locations. Previous research from Marrakech identified gastric cancer as the most common digestive cancer in the city which helps to verify our results. Both studies identified an average age of diagnosis of 59 years; and 64–70% of all cases were in males [17].

The significant difference in gastric cancer incidence found between Marrakech and Casablanca is the first to be reported for these cities. However, similar trends have been reported in South and Central America. A 25-time higher rate was found in a town in the Andes Mountains in Colombia compared to the rate in a city that is 200 kilometers away on the coast. The difference in rates was attributed to a mismatch in the ancestry of the host and the microbe, which resulted in more carcinogenic gastric lesions in the high rate area [18]. Similar differences were reported in Chile and Guatemala where higher gastric cancer rates were found among higher altitudes [19]. Increased gastric cancer risks associated with environment and genotypic variations of the* H. pylori* strains have been well documented and these factors may explain the variations in gastric cancer rates in populations that have nearly identical infection rates [20–22].

Inclusion of data from the major cancer hospital and the main private pathology laboratories in Marrakech assured the comprehensiveness of data sources. Obtaining records from the largest private pathology laboratories andpublic hospital in the city allowed for a wider representation of socioeconomic backgrounds of patients and increased inclusion of the majority of gastric cancer patients in the region. The existence of census information for the population and age and sex strata allowed us to calculate incidence, age-specific, and sex-specific rates for Marrakech. Availability of a quality population-based cancer registry in Casablanca and the opportunity to compare its results to Marrakech increased the efficiency of region comparison of rates in this study.

Lack of information about the residence of the patients from the private pathology laboratories limited our ability to calculate region-specific rates. However, there is no reason to believe that patients from distant regions of Morocco would seek histopathologic diagnosis in these laboratories in Marrakech. Due to possible cultural norms of not seeking medical care or limited availability of health professionals in peripheral regions, there is a possibility of underestimation of gastric cancer from these regions [23]. One of the main focal points of the study was to examine the most recent 5-year period of gastric cancer in Marrakech rather than choose a previous matching period with the Casablanca cancer registry. The reason was that the new cancer center in Marrakech was opened in the 5 years preceding the study and the flow of patients was much larger and inclusive. Also, we are not aware of significant changes in the healthcare or exposure factors related to gastric cancer that occurred in Marrakech between the 2 data intervals of 2004–2007 and 2008–2012. However, this nonoverlapping period might be a limitation of the study.

A unique mix of cultural and lifestyle factors and regional differences in Morocco present an opportunity for future research into the investigation of risk factors that may contribute to the higher gastric cancer rate in Marrakech. Previous research has shown that risk factors for gastric cancer could be categorized into (a)* H. pylori* infection [24], (b) nitrogen compounds and other chemical carcinogens [25], (c) high intake of salted, smoked, and cured foods [2, 26], and (d) other social and behavioral factors [25, 27–29]. With scarce current data from Morocco, these risk factors should be investigated in future studies in Marrakech and other parts of Morocco to elucidate the risk factors of gastric cancer in this population.

## 5. Conclusions

In conclusion, a significantly higher rate of gastric cancer was found in Marrakech when compared to the rates of Casablanca. Notably, the difference in incidence in males between Marrakech and Casablanca was much more distinct than the difference in females. The incidence of the disease in Marrakech could be higher than what is reported in this study because of the possible missed patients who did not seek medical care and die without diagnosis or documentation. Future studies should further evaluate the possible underestimation of gastric cancer in Marrakech and explore the variable rates in other regions of Morocco. Future studies should also explore the risk factors of the disease including infectious, dietary, and environmental factors and possible regional differences.

## Figures and Tables

**Table 1:** Demographic information of the study population gastric cancer patients in Marrakech in 2008–2012.

Variable	Number	%
Age (years), all (*n* = 725)		
Median	*60*	
Range	*19*–*94*	
Age (years), males		
Median	*62*	
Range	*19*–*94*	
Age (years), females		
Median	*58*	
Range	*23*–*88*	
Sex (*n* = 725)		
Male	467	64
Female	258	36
^a^Residence (*n* = 292)		
Urban/semiurban	232	79
Rural	48	16
^a^Diagnostic information (*n* = 292)		
Endoscopy	252	86
CT scan	237	81

^a^ *n* = 292, as this represents the patients included from the medical records from the hospital. Residence and diagnostic information was only found in these records.

**Table 2:** Histopathologic information of gastric cancer patients in Marrakech in 2008–2012.

Variable	Number	%
Pathology (*n* = 725)		
Biopsy	655	90
Resection	111	15
Histological type (*n* = 725)		
Adenocarcinoma	602	83
Carcinoma	91	13
^a^GIST	22	3
Not given	10	1
^b^Differentiation (*n* = 725)		
Well	39	5
Moderately	288	38
Poorly	281	39
Undifferentiated	17	2
Not specified	78	11

^a^GIST: gastrointestinal stromal tumor.

^b^The sum of percentages for differentiation does not total 100% because cases of GIST are not reported with level of differentiation.

**Table 3:** Age-specific incidence rates in Marrakech (2008–2012) and Casablanca (2004–2007).

	Marrakech	Casablanca	Rate ratio (95% CI)
<19	0.019	0.159	**0.1** (*0.02–0.96*)
20–24	0.078	0.274	**0.3** (*0.03–2.56*)
25–34	1.71	1.50	**1.1** (*0.73–1.78*)
35–44	4.22	4.08	**1.0** (*0.76–1.40*)
45–54	9.05	11.3	**0.8** (*0.64–1.01*)
55–64	30.0	21.8	**1.4** (*1.13–1.68*)
65–74	41.1	40.9	**1.0** (*0.82–1.23*)
>75	39.7	34.0	**1.2** (*0.88–1.54*)

**Table 4:** Age-specific incidence rates for males in Marrakech (2008–2012) and Casablanca (2004–2007).

	Marrakech	Casablanca	Rate ratio (95% CI)
<19	0.038	0.079	**0.5** (*0.04–5.32*)
20–24	0.164	0.138	**1.2** (*0.07–18.88*)
25–34	1.61	0.539	**3.0** (*1.24–7.21*)
35–44	5.23	3.10	**1.7** (*1.08–2.64*)
45–54	10.2	7.22	**1.4** (*1.00–1.98*)
55–64	39.2	18.2	**2.2** (*1.62–2.86*)
65–74	64.5	28.2	**2.3** (*1.70–3.09*)
>75	53.1	24.3	**2.2** (*1.43–3.35*)

**Table 5:** Age-specific incidence rates for females in Marrakech (2008–2012) and Casablanca (2004–2007).

	Marrakech	Casablanca	Rate ratio (95% CI)
<19	0.0	0.080	**0.0**
20–24	0.0	0.271	**0.0**
25–34	1.81	1.24	**1.5** (*0.77–2.76*)
35–44	3.29	2.15	**1.5** (*0.90–2.59*)
45–54	7.80	4.78	**1.6** (*1.08–2.48*)
55–64	21.5	7.79	**2.8** (*1.84–4.15*)
65–74	20.1	17.6	**1.1** (*0.76–1.71*)
>75	26.5	12.6	**2.1** (*1.20–3.71*)

**Table 6:** Total incidence comparison in Marrakech (2008–2012) and Casablanca (2004–2007).

	Marrakech	Casablanca	Rate ratio
Male	7.19	3.91	**1.8** (*1.6–2.1*)
Female	3.87	2.58	**1.5** (*1.2–1.8*)
All	5.50	3.23	**1.7** (*1.5–1.9*)
